# Methylmercury induces neuronal cell death by inducing TNF-α expression through the ASK1/p38 signaling pathway in microglia

**DOI:** 10.1038/s41598-021-89210-7

**Published:** 2021-05-10

**Authors:** Takashi Toyama, Takayuki Hoshi, Takuya Noguchi, Yoshiro Saito, Atsushi Matsuzawa, Akira Naganuma, Gi-Wook Hwang

**Affiliations:** 1 Laboratory of Molecular and Biochemical Toxicology, Graduate School of Pharmaceutical Sciences, Tohoku University, 6-3 Aoba, Aoba-ku, Aramaki, Sendai, Miyagi 980-8578 Japan; 2 Laboratory of Molecular Biology and Metabolism, Graduate School of Pharmaceutical Sciences, Tohoku University, 6-3 Aoba, Aoba-ku, Sendai, Miyagi 980-8578 Japan; 3 Laboratory of Environmental and Health Sciences, Faculty of Pharmaceutical Sciences, Tohoku Medical and Pharmaceutical University, 4-4-1 Komatsushima, Aoba-ku, Sendai, Miyagi 981-8558 Japan; 4 Laboratory of Health Chemistry, Graduate School of Pharmaceutical Sciences, Tohoku University, 6-3 Aoba, Aoba-ku, Aramaki, Sendai, Miyagi 980-8578 Japan

**Keywords:** Mechanism of action, Metals

## Abstract

We recently found that tumor necrosis factor-α (TNF-α) may be involved in neuronal cell death induced by methylmercury in the mouse brain. Here, we examined the cells involved in the induction of TNF-α expression by methylmercury in the mouse brain by in situ hybridization. TNF-α-expressing cells were found throughout the brain and were identified as microglia by immunostaining for ionized calcium binding adaptor molecule 1 (Iba1). Methylmercury induced TNF-α expression in mouse primary microglia and mouse microglial cell line BV2. Knockdown of apoptosis signal-regulating kinase 1 (ASK1), an inflammatory cytokine up-regulator that is responsible for reactive oxygen species (ROS), decreased methylmercury-induced TNF-α expression through decreased phosphorylation of p38 MAP kinase in BV2 cells. Suppression of methylmercury-induced reactive oxygen species (ROS) by antioxidant treatment largely abolished the induction of TNF-α expression and phosphorylation of p38 by methylmercury in BV2 cells. Finally, in mouse brain slices, the TNF-α antagonist (WP9QY) inhibited neuronal cell death induced by methylmercury, as did the p38 inhibitor SB203580 and liposomal clodronate (a microglia-depleting agent). These results indicate that methylmercury induces mitochondrial ROS that are involved in activation of the ASK1/p38 pathway in microglia and that this is associated with induction of TNF-α expression and neuronal cell death.

## Introduction

Methylmercury is an environmental contaminant that bioaccumulates in fish and shellfish. When methylmercury is taken into the human body, it binds with free cysteine to form a methionine-like structure that is then transported to the brain and fetus via the neutral amino acid transporter (LAT1 and LAT2)^1^. Excessive intake of methylmercury can cause Minamata disease and fetal Minamata disease, which are both serious central nervous system disorders^2,3^. Methylmercury is widespread in the environment and is produced by the methylation of inorganic mercury in the environment by microorganisms. In recent years, epidemiological studies have suggested that excessive consumption of fish and shellfish containing high levels of methylmercury during pregnancy may have adverse effects on the motor function and intellectual development of the child^4–7^. However, the mechanisms involved in methylmercury-induced central nervous system damage remain unclear.

Intercellular crosstalk between neurons and astrocytes or microglia has been shown to be important for neuronal damage and neuroprotection^8–10^. Microglia and astrocytes activate neuropathic or protective microglia or astrocytes, which release signaling agents such as pro-inflammatory cytokines and chemokines^11–13^. Previously, Rosten et al*.* found that the chemokine C–C Motif Chemokine Ligand (CCL) 2 suppresses methylmercury-induced neuronal damage in primary rat neurons^14^. Koizumi et al*.* reported that methylmercury-induced interleukin-6 (IL-6) expression in astrocytes suppresses methylmercury-induced neuronal cell death^15,16^. Induction of methylmercury-induced IL-6 expression in the astrocytes in the midbrain of mice has been shown to be protective against methylmercury-induced hearing impairment^17^. Furthermore, we found that methylmercury-induced expression of the chemokine CCL4 in the mouse brain suppresses neuronal damage^18^. However, the cytokines and chemokines involved in methylmercury toxicity are not well known, although some cytokines and chemokines have been reported to exert protective effects against methylmercury-induced neuronal damage. We have previously found that methylmercury selectively induces expression of tumor necrosis factor-α (TNF-α) in the mouse brain^19^, where relatively low concentrations of methylmercury accumulate, in contrast to the liver and kidneys^19^. The addition of the recombinant TNF-α to cultured mouse neural stem cells enhances methylmercury toxicity, which strongly suggests that the induction of TNF-α expression in the central nervous system by methylmercury plays a role in neuronal damage^19^. Shinoda et al*.* also reported that methylmercury induces neuronal damage in the spinal dorsal root ganglia of rats treated with methylmercury, accompanied by the induction of TNF-α expression, an inflammatory cytokine^20^. TNF-α binds to TNF receptor 1 (TNFR1) and recruits TNF receptor-associated factor (TRAF) molecules to its intracellular domain^21^, which leads to neuronal cell death by caspase-8-mediated apoptosis and receptor-interacting serine/threonine-protein kinase 1(RIP1)-mediated necroptosis^22^. In neurodegenerative diseases such as Alzheimer's disease and amyotrophic lateral sclerosis, the release of TNF-α from microglia and astrocytes is also known to play a role in disease progression^23–25^. Therefore, we hypothesized that elucidating the mechanism of TNF-α expression induced by methylmercury may help to understand the mechanisms underlying methylmercury-induced central nervous system damage. In this study, we identified the cells responsible for the induction of TNF-α expression in mouse brains treated with methylmercury, with the aim of determining the mechanism of TNF-α expression.

Apoptosis signal-regulating kinase 1 (ASK1), a mitogen-activated protein (MAP) kinase (MAP3K) family member, is activated in response to various stresses, including oxidative stress, pathogens, calcium overload, and endoplasmic reticulum stress, and activates both the c-Jun N-terminal kinase (JNK) and p38 MAP kinase pathways^26,27^. Among the various stimuli that activate ASK1, reactive oxygen species (ROS) are one of the most potent activators of ASK1, and the mechanisms by which ROS activate ASK1 are well characterized. ASK1 constitutively forms a high molecular mass complex (1,500–2,000 kDa) with ASK1 regulators including thioredoxin (Trx), a redox-sensing protein that inhibits the kinase activity of ASK1 by directly binding to the N-terminal noncatalytic region of ASK1 ^28^. In the presence of ROS, Trx is oxidized and dissociated from the ASK1 signaling complex, and reciprocally, TRAF2 and TRAF6 are recruited to the ASK1 signaling complex, which are required for ROS-dependent ASK1 activation. ROS-dependent activation of ASK1 mediates multiple cellular responses including inflammation and cell death through the activation of p38 MAP kinases (MAPK)^29,30^. Thus, the ASK1-p38 axis functions as an essential component of redox signaling. Meanwhile, transforming growth factor-β (TGF-β)-activated kinase 1 (TAK1) is also an ROS-responsive kinase that belongs to the MAP3K family^31,32^. In addition to the JNK and p38 MAP kinase pathways, TAK1 can activate the nuclear factor (NF)-κB and Kelch-like ECH-associated protein 1–nuclear factor (erythroid-derived 2)-like 2 (Keap1-Nrf2) pathways that promote cell survival, which impacts the functional output when compared with ASK1 and induces cell death^32,33^.

Here, we report that methylmercury induces TNF-α expression in microglia in the mouse brain. This is triggered by the ASK1/p38 pathway, which is a redox signal that is involved in the inflammatory responses.

## Results

### Identification of the cells involved in the induction of TNF-α mRNA in the brains of mice administered with methylmercury

**Figure 1:**
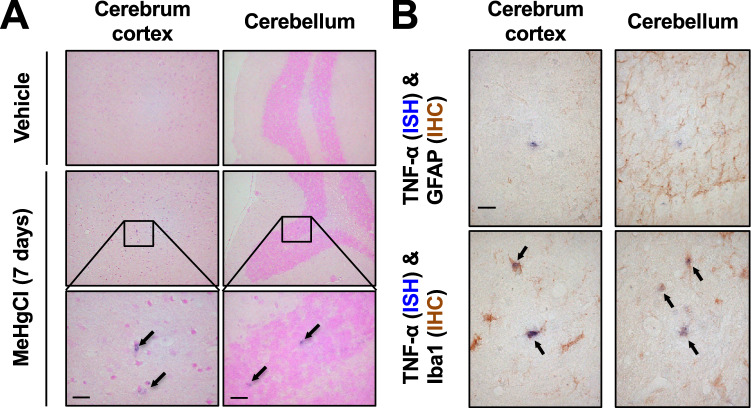
TNF-α expressing cells in the brains of mice treated with methylmercury. (**A**) Mice were injected with methylmercuric chloride (MeHgCl; 25 mg/kg) by S.C. and kept for 7 days *ad libitum*. The brains were subjected to in situ hybridization for TNF-α mRNA. Representative images are shown. Black arrows indicate TNF-α expressing cells. (**B**) The section was immunostained for GFAP or Iba1 antibodies. Black arrows indicate double positive cells of TNF-α and Iba1. Scale bars indicate 25 µm.

**Figure 2:**
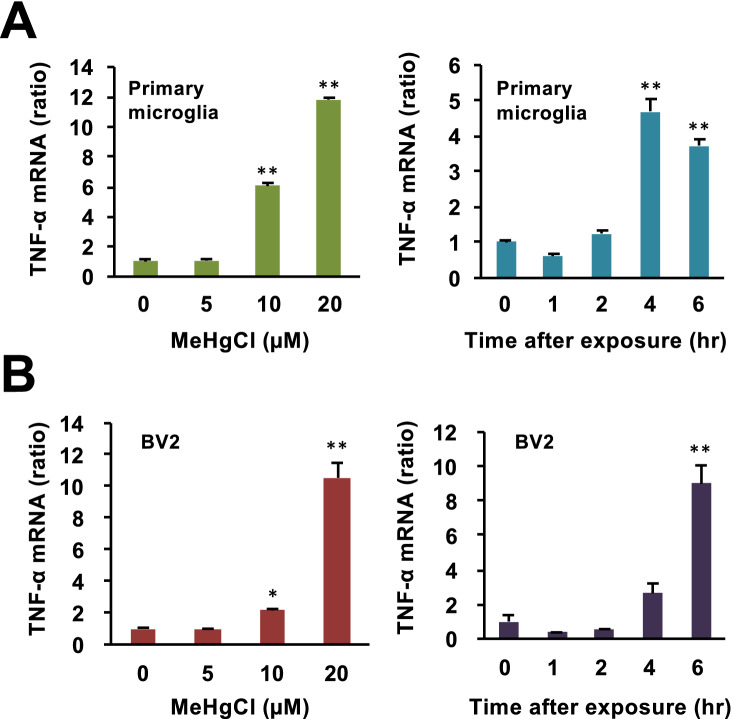
Effects of methylmercury on TNF-α expression in primary microglia and BV2 cells. (**A**) Mouse primary microglia were exposed to indicated concentrations of MeHgCl for 6 h (left panel) or 10 µM of MeHgCl for the indicated time course (right panel). The data are presented as GAPDH-corrected means ± standard deviations. (**B**) Mouse microglial cell lines (BV2) were exposed to indicated concentrations of MeHgCl for 6 h (left panel) or 20 µM of MeHgCl for the indicated time course (right panel). mRNA levels of TNF-α were measured by qPCR (n = 3). The data are presented as actin-corrected means ± standard deviations. The Y-axis indicates the ratio with the control as 1. *P < 0.05 vs control. **P < 0.01 vs control.

We previously reported that methylmercury induces TNF-α expression in the cerebral cortex and cerebellum of mice in a time-dependent manner^19^. Therefore, we subcutaneously administered methylmercury to mice (25 mg/kg for 7 days) under the same conditions as those in which TNF-α expression was markedly induced, and the obtained brains were used for in situ hybridization against TNF-α mRNA. In the control mice, which were subcutaneously treated with saline, TNF-α-expressing cells were not found, whereas in the methylmercury-treated group, TNF-α-expressing cells were found in the cerebral cortex and cerebellum (Fig. 1A). Under our study conditions, methylmercury accumulation in the cortex and the cerebellum was comparable (Supplemental Fig. 1). Because both astrocytes and microglia may be involved in the induction of TNF-α expression in the mouse brain^34,35^, we performed immunostaining using antibodies against glial fibrillary acidic protein (GFAP) or ionized calcium binding adaptor molecule 1 (Iba1), which are proteins specifically expressed in astrocytes and microglia, respectively^36,37^. We found that the TNF-α expressing cells did not overlap with the GFAP antibody stained images but did with the Iba1 antibody stained images (Fig. 1B and Supplemental Fig. 2). This suggests that the induction of TNF-α expression by methylmercury in the mouse brain mainly involves microglia. We next treated the mouse primary microglia and mouse microglial cell line, BV2, with methylmercury and found that TNF-α expression was induced in a concentration- and time-dependent manner (Fig. 2A, B). These results suggest that methylmercury may act directly on microglia to induce TNF-α expression. When BV2 cells were treated with at least 30 µM of methylmercury for more than 6 h or 20 µM of methylmercury for more than 8 h, cell adhesion and actin mRNA were reduced (Supplemental Fig. 3A–C). Therefore, in the subsequent experiments, we treated BV2 cells with 20 µM methylmercury for 6 h as the optimal treatment condition in which methylmercury induces TNF-α expression.

**Figure 3:**
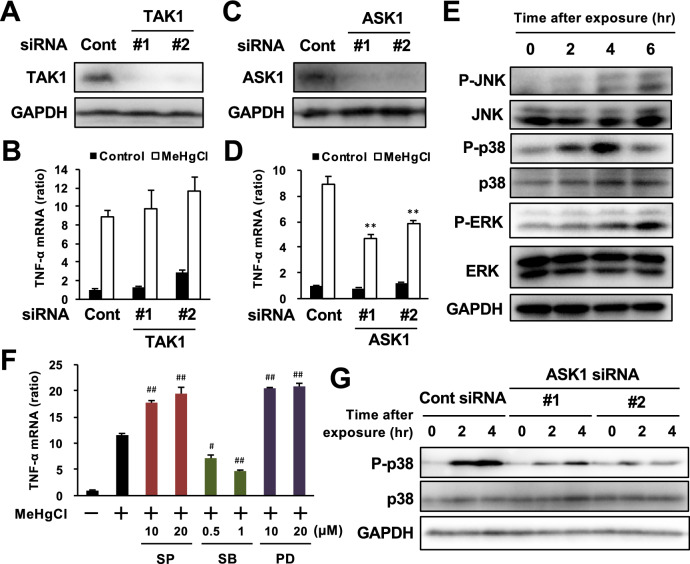
Involvement of MAP kinase pathways in methylmercury-induced TNF-α expression in BV2 cells. BV2 cells were transfected with two different sequences of TAK1 or ASK1 siRNA (#1 or #2) and incubated for 24 h or 48 h (there was no suppression of ASK1 expression at 24 h after siRNA transfection). Protein levels of (**A**) TAK1 or (**C**) ASK1 were determined by Western blotting. (**B,D**) The cells were exposed to MeHgCl (20 µM) for 6 h and mRNA levels of TNF-α were measured by qPCR (n = 3). The data are shown as actin-corrected means ± standard deviations. The Y-axis indicates the ratio with the control as 1. (**E**) BV2 cells were exposed to 20 µM of MeHgCl for the indicated time course and phosphorylation of MAP kinases were determined by Western blotting. (**F**) The cells were pretreated with indicated concentrations of SP600125 (SP; JNK inhibitor), SB203580 (SB; p38 inhibitor), or PD98059 (PD; MEK/ERK inhibitor) for 30 min, then 20 µM of MeHgCl were added to the medium and incubated for a further 6 h. mRNA levels of TNF-α were measured by qPCR (n = 3). The data are shown as actin-corrected means ± standard deviations. The Y-axis indicates the ratio with the control as 1. (**G**) The cells were transfected with two different sequences of ASK1 siRNA for 48 h. The cells were exposed to 20 µM of MeHgCl for the indicated time course and phosphorylation of p38 was determined by Western blotting. *P < 0.05 vs control, **P < 0.01 vs control. ^#^ < 0.05 vs MeHgCl(+), ^##^ < 0.01 vs MeHgCl(+).

### Signaling pathways involved in methylmercury-induced TNF-α expression in microglia

TAK1 and ASK1 are known to be involved in the induction of TNF-α expression in microglia as MAP3Ks^38–40^. Therefore, we investigated the involvement of TAK1 and ASK1 in the induction of TNF-α expression by methylmercury using two different small interfering RNAs (siRNA) against them. Because the transfection of siRNA into primary microglia was failed, we examined the effect of methylmercury on the induction of TNF-α expression by transfecting BV2 cells with each siRNA. The protein levels of TAK1 and ASK1 in BV2 cells were reduced by each siRNA (Fig. 3A, C). When cells were treated with methylmercury under these conditions, the induction of TNF-α expression was significantly reduced by the suppression of ASK1 but almost unaffected by the suppression of TAK1 (Fig. 3B, D). Because ASK1 may induce TNF-α expression through phosphorylation of MAPKs, we examined the phosphorylation of representative MAPKs, JNK, p38, and extracellular signal-related kinase (ERK), and found that all MAPKs were phosphorylated in a methylmercury treatment time-dependent manner (Fig. 3E). Conversely, when the cells were treated with each of the inhibitors to their respective MAPKs, the induction of TNF-α expression by methylmercury was significantly reduced only by treatment with SB203580 (an inhibitor of p38) but increased with ERK and JNK inhibitors (Fig. 3F). Treatment with another p38 inhibitor, SB202190, also reduced the induction of TNF-α expression by methylmercury (Supplemental Fig. 4), which suggests that p38 contributes to methylmercury-induced TNF-α expression. Furthermore, the phosphorylation of p38 by methylmercury was markedly inhibited by ASK1 knockdown (Fig. 3G), which indicates that methylmercury induces TNF-α expression in microglia via the ASK1/p38 pathway.

**Figure 4:**
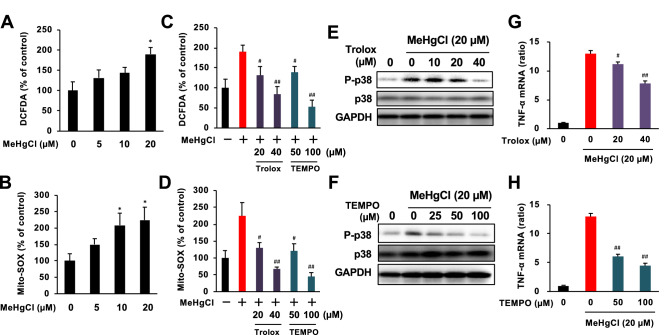
Effects of antioxidants on the induction of TNF-α expression by methylmercury via the ASK1/p38 pathway in BV2 cells. BV2 cells were loaded with (**A**) 10 µM of H_2_DCF-DA or (**B**) 1 µM of Mito-SOX for 30 min. The cells were exposed to indicated concentrations of MeHgCl for 6 h and fluorescence was determined by (**A**) intracellular ROS or (**B**) mitochondrial ROS (mtROS) levels. The cells were pretreated with indicated concentrations of Trolox or Mito-TEMPO (TEMPO) for 30 min and exposed to 20 µM of MeHgCl for 6 h and fluorescence was determined by (**C**) intracellular ROS or (**D**) mtROS levels. (**E,F**) Phosphorylation of p38 was determined by Western blotting. (**G,H**) mRNA levels of TNF-α were measured by qPCR (n = 3). The data are shown as actin-corrected means ± standard deviations. The Y-axis indicates the ratio with the control as 1. *P < 0.05 vs control, *P < 0.01 vs control. ^#^ < 0.05 vs MeHgCl(+), ^##^ < 0.01 vs MeHgCl(+).

### Involvement of oxidative stress on methylmercury-induced p38 activation and TNF-α expression in microglia

Under normal conditions, the activity of ASK1 is inhibited by binding to Trx. However, when Trx forms intramolecular disulfide bonds upon oxidative stress, ASK1 dissociates and promotes autophosphorylation^41^. We therefore examined whether ROS were involved in the activation of ASK1 by methylmercury. We first measured intracellular ROS levels by 2′,7′-dichlorodihydrofluorescein diacetate (H_2_DCF-DA) and found that methylmercury significantly increased ROS levels under conditions in which TNF-α expression was induced (Fig. 4A). ROS is known to be produced via nicotinamide adenine dinucleotide phosphate oxidase and nitric oxide synthase in immunocompetent cells, such as microglia, macrophages and monocytes^42–45^. However, methylmercury is known to produce ROS through mitochondrial damage^46–48^. Therefore, when ROS levels in mitochondria (mtROS) were examined in Mito-SOX, the levels had increased in a methylmercury concentration-dependent manner (Fig. 4B). This increase was significantly suppressed by treatment with the antioxidant, Trolox, and was similarly reduced to basal levels by treatment with the mitochondria-specific ROS scavenger, Mito-TEMPO (Fig. 4C). Furthermore, the mtROS levels that were increased by methylmercury were completely suppressed by Mito-TEMPO treatment and reduced to basal levels by Trolox treatment (Fig. 4D). These results suggest that in microglia, methylmercury increases ROS mainly through mitochondria. Additionally, Trolox and Mito-TEMPO treatment inhibited the phosphorylation of p38 by methylmercury (Fig. 4E, F) and similarly suppressed the induction of TNF-α expression (Fig. 4G, H), which indicates that methylmercury induces TNF-α expression via the mtROS/ASK1/p38 pathway.

### Involvement of the NF-κB pathway in methylmercury-induced TNF-α expression via the ASK1/p38 pathway

**Figure 5:**
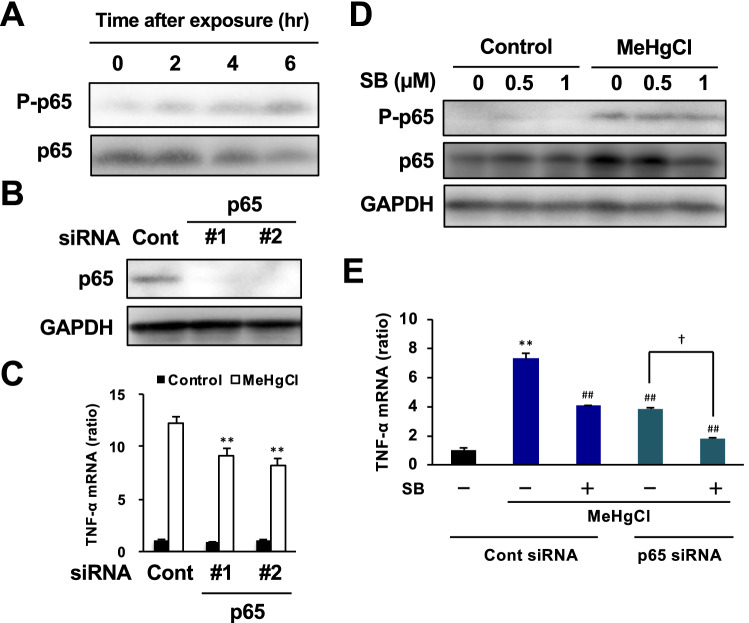
Involvement of NF-κB signaling in methylmercury-induced TNF-α expression via p38 in BV2 cells. (**A**) BV2 cells were exposed to MeHgCl (20 µM) for the indicated time course, then Western blotting was performed. (**B**) Cells were transfected with two different sequences of p65 siRNA (#1 or #2) and incubated for 24 h. Protein levels of p65 were determined by Western blotting. (**C**) The cells were exposed to MeHgCl (20 µM) for 6 h and mRNA levels of TNF-α were measured by qPCR (n = 3). (**D**) The cells were pretreated with indicated concentrations of SB203580 (SB; p38 inhibitor) for 30 min, then 20 µM of MeHgCl were added to the medium and incubated for a further 4 h, followed by Western blotting. (**E**) After the exposure of MeHgCl for 6 h, mRNA levels of TNF-α were measured by qPCR (n = 3). All qPCR data are shown as actin-corrected means ± standard deviations. The Y-axis indicates the ratio with the control as 1. *P < 0.01 vs control. ^##^ < 0.01 vs SB(−) MeHgCl(+). ^†^ < 0.01 vs SB(−) p65 siRNA + MeHgCl(+).

We previously showed that activation of NF-κB by methylmercury contributes to the induction of TNF-α expression in cultured mouse neural stem cells^19^. In addition to determining the mechanism of induction of TNF-α expression via the ASK1/p38 pathway in microglia, we also examined the involvement of the NF-κB pathway. Treatment of BV2 cells with methylmercury increased time-dependent phosphorylated levels of p65, a subunit of NF-κB, which peaked at 4 h (Fig. 5A). Knockdown of p65 reduced the induction of TNF-α expression by methylmercury (Fig. 5B, C), which indicated that activation of the NF-κB pathway by methylmercury is also involved in the induction of TNF-α expression in microglia. However, the p38 inhibitor did not affect the activation of the NF-kB pathway by methylmercury; this inhibitor further reduced methylmercury-induced TNF-α expression, which was already reduced by the knockdown of p65 (Fig. 5D, E). These suggest that the NF-κB and ASK1/p38 pathways may be independently involved in the induction of TNF-α expression by methylmercury. Furthermore, we found that treatment with an IκB kinase (IKK) inhibitor (BAY11-7082) under conditions that inhibited the activation of p65 by lipopolysaccharide did not inhibit the phosphorylation and nuclear translocation of p65 by methylmercury (unpublished observation). This suggests that methylmercury activates the NF-κB pathway via a different pathway to the canonical signaling pathway, which requires more detailed examination in future studies.

### Involvement of microglial p38 activation in neuronal cell death, induced by methylmercury in mouse brain slices

**Figure 6:**
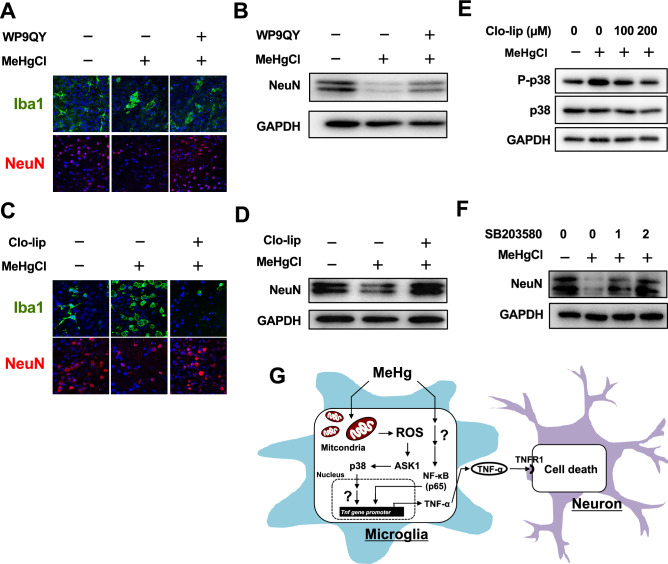
Role of microglial p38 activation in methylmercury-induced neuronal cell death. (**A**) Mouse brain slices were treated with 10 µM of TNF-α antagonist (WP9QY) for 72 h. The slices were exposed to 50 µM of MeHgCl for 24 h and immunostained for Iba1 (green) or NeuN (red). (**b**) Western blotting of NeuN was performed in the same conditions as in A. (**C**) Mouse brain slices were treated with 100 µM of liposomal clodronate (Clo-lip) for 72 h. The slices were exposed to 50 µM of MeHgCl for 24 h and immunostained for Iba1 (green) or NeuN (red). (**D**) Western blotting of NeuN was performed in the same conditions as in C. (**E**) Six hours after exposure to MeHgCl, Western blotting of P-p38 and p38 were performed. (**F**) Indicated concentrations of SB203580 (SB; p38 inhibitor) were pretreated for 30 min and the slices were exposed to 50 µM of MeHgCl for 24 h, followed by Western blotting of NeuN. (**G**) The mechanism underlying methylmercury-induced neuronal cell death through the induction of TNF-α expression.

Then we examined the effects of the induction of TNF-α expression via activation of p38 in microglia on neuronal cell death. We previously found that methylmercury-induced TNF-α expression can induce neuronal cell death through its release into the medium^19^. Therefore, we first treated the mouse brain slices with methylmercury and found that the number of cells positive for the microglial marker, Iba1, increased significantly (microglial activation), while the number of cells positive for the neuronal marker, NeuN, decreased (Fig. 6A). Pretreatment of mouse brain slices with WP9QY, which inhibits the binding of TNF-α to its receptor^49^, suppressed the decrease in NeuN-positive cells by methylmercury. Similar results were also obtained by Western blotting with the NeuN antibody (Fig. 6B). These findings suggest that TNF-α is released from the microglia that are activated by methylmercury, inducing neuronal cell death. Liposomal clodronate is known to selectively eliminate microglia in the brain; it is specifically taken up by phagocytes and induces cell death^50^. Treatment of the mouse brain slices with liposomal clodronate markedly suppressed the observed increase in Iba1 by methylmercury (Fig. 6C). Treatment of mouse brain slices with methylmercury reduced the levels of NeuN and thus indicated neuronal cell death, which was largely inhibited by liposomal clodronate treatment (Fig. 6D). Additionally, under the same conditions, liposomal clodronate inhibited the activation of p38 by methylmercury, which suggests that methylmercury may have activated p38 in the microglia in these brain slices (Fig. 6E). Furthermore, treatment of mouse brain slices with a p38 inhibitor prevented the methylmercury-induced reduction of the neuronal marker, NeuN (Fig. 6F), which suggests that methylmercury may induce neuronal cell death by activating p38 in microglia.

## Discussion

The present study revealed that methylmercury induced TNF-α expression in microglia in the mouse brain, which was due to the activation of the ASK1/p38 pathway via mtROS production. The findings also suggest that these pathways are involved in neuronal cell death caused by methylmercury. However, it has been reported that sub-µM order of methylmercury can cause neuronal damage, thus methylmercury concentrations used in this study is relatively high and it is difficult to extrapolate the phenomenon observed in vivo and other studies. Although this is a limitation of this study, the present results clearly indicate that microglia are involved in methylmercury-induced TNF-α expression.

Induction of TNF-α in astrocytes and microglia is known to be involved in various neurological disorders associated with inflammation^34,51^. Although methylmercury has been reported to increase ROS production in astrocytes^34,35^, the present study revealed that methylmercury increased TNF-α expression via the mtROS/ASK1/p38 pathway in a microglia-specific manner. However, we did not find any evidence that might provide clues as to whether microglia specifically respond to methylmercury to induce TNF-α expression. It has been reported that microglia have lower glutathione levels than astrocytes^52–54^, which also results in higher production of ROS by methylmercury^55^. These suggest that microglia may be more sensitive to oxidative stress than astrocytes. Therefore, it is possible that the induction of selective TNF-α expression in microglia involves differences in sensitivity to oxidative stress induced by methylmercury.

In addition, since relatively high concentrations of methylmercury were found to induce TNF-α expression in this study, the possibility that methylmercury-induced release of damage-associated molecular patterns (DAMPs) from neurons may be involved in TNF-α induction in vivo and ex vivo cannot be denied, and further studies are needed to clarify the mechanisms.

It has been shown that methylmercury increases mtROS production and decreases adenosine triphosphate production by inhibiting the mitochondrial electron transfer system^56^. Previously, we reported that in human neuroblastoma cells treated with methylmercury, pyruvate accumulates in mitochondria as a result of a decrease in the mitochondrial membrane potential, which is involved in methylmercury toxicity via an increase in mtROS production^48^. However, in microglia, this increase in mtROS production was observed before methylmercury-induced neuronal cell death, which suggests that it is involved in the activation of the signaling pathway for the induction of TNF-α expression. Indeed, the increase in ROS production upon induction of toll-like receptor 4-mediated TNF-α expression by pro-inflammatory ligands, such as lipopolysaccharides (LPS) in macrophages, combined with the fact that the release of oxidized mitochondrial DNA associated with mtROS production contributes to the inflammatory response and activation of inflammasomes^57^, suggest that the increase in mtROS levels by methylmercury may be a physiological inflammatory response in microglia, which are immune cells in the brain. The ROS produced in microglia activate ASK1 by oxidizing Trx (a negative regulator of ASK1) and p38 via the MAPK cascade^28^. Because antioxidant treatment inhibits the phosphorylation of p38 by methylmercury, the activation of the ASK1/p38 pathway by methylmercury is expected to occur by a similar mechanism. Given that we now understand that increased ROS levels in microglia and macrophages play an important role in the induction of proinflammatory cytokine expression, the future aim is to determine the mechanism underlying the increased mtROS production in microglia by methylmercury. Interestingly, Fujimura et al., recently reported that inflammatory activation of microglia by methylmercury via Rho/ROCK pathway is crucial for methylmercury-induced axonal degeneration *in vivo*^58^. ROS are known as driver of Rho/ROCK pathway^59,60^, thus further studies are need to elucidate the signaling underlying ROS-mediated TNF-α induction in microglia. In addition to the previous studies, this study will enable us to understand the mechanisms involved in neurotoxicity through proinflammatory cytokine-mediated crosstalk between microglia and neurons.

The induction of TNF-α expression by methylmercury was completely inhibited by treatment with Actinomycin D, a transcriptional inhibitor (Supplemental Fig. 5), which suggests that NF-κB and downstream transcription factors of p38 are involved in the induction of TNF-α expression. p38 is known to activates various transcription factors, such as signal transducer and activator of transcription 1 (STAT1), activating transcription factor 2 (ATF2), and c-Fos^61–63^. It has been reported that in macrophages, STAT1 is involved in the induction of TNF-α expression by binding to NF-kB^64^, while in activated T cells, ATF2 is involved in the induction of TNF-α expression by binding to c-Jun^65^. c-Fos is also known to form heterodimers with c-Jun and induce TNF-α expression as a transcription factor activator protein 1 ^66,67^. We expected these transcription factors to be involved in the methylmercury-induced TNF-α expression via p38. Although methylmercury activated both c-Fos and c-Jun in the BV2 cells (Supplemental Fig. 6), knockdown of either factor did not reduce the induction of TNF-α expression by methylmercury; this was the case even under simultaneous knockdown of both factors (Supplemental Fig. 7). Therefore, the transcription factors involved in the induction of TNF-α expression in the downstream factor of p38 that are activated by methylmercury remain unclear and require further investigation.

In mouse brain slices, methylmercury was shown to induce neuronal cell death via the activation of p38 in microglia. Because the IKK inhibitor (BAY11-7082) failed to inhibit p65 phosphorylation by methylmercury, as mentioned above (unpublished observation), we were unable to examine the contribution of NF-κB in the neuronal cell death induced by methylmercury. The result shown in Fig. 6G suggests that methylmercury induces TNF-α expression via p38 activation and that TNF-α is released extracellularly and binds to TNFR1 on the neuronal cell membrane, which induces cell death^68^. It has been reported that colony stimulating factor-1 activates p38 to release brain-derived neurotrophic factor from microglia, which is involved in the enhancement of neuronal pain-sensing that is caused by the calcitonin gene-related peptide^69^. Microglia are also known to cause cell death and mitochondrial dysfunction in neuronal cells via the release of interleukin-1β by activating p38 in response to amyloid β^70,71^. Additionally, activators of nuclear factor erythroid 2-related factor 2, a regulator of the antioxidant system, have been shown to inhibit LPS-induced neuronal cell death by suppressing p38 activation in microglia^72^. Thus, activation of p38 in microglia may be an important signaling pathway that mediates not only methylmercury, but also neuroinflammation and neuronal cell death caused by diverse inflammatory stimuli and pathologies. Future investigations of the mechanism of p38 activation by methylmercury in microglia using mouse brain slice cultures will not only elucidate the mechanism of methylmercury-induced neuronal cell death, but also provide valuable knowledge toward understanding the mechanisms that underlie neurological damage that is caused by inflammatory responses in the brain.

## Materials and methods

### Animals

All mice used in the study were C57BL/6 (6 weeks old, male) that were purchased from Japan SLC Inc. (Shizuoka, Japan). The mice were housed in plastic cages at 22 ± 2 ℃ and a relative humidity of 55 ± 20%, with a 12-h light–dark cycle. Food (F-2, Oriental Yeast, Tokyo, Japan) and tap water were given freely to the mice. Mice were kept for 1 week for adaptation, subcutaneously injected with methylmercuric chloride (MeHgCl; 25 mg/kg) dissolved in saline and dissected after the indicated time period. The present study was performed in compliance with the ARRIVE guidelines and accordance with the Regulations for Animal Experiments and Related Activities at Tohoku University.

### In situ hybridization and immunohistochemistry

The dissected mice were perfused with G-Fix from the left ventricle (GenoStaff, Tokyo, Japan). Isolated brains were soaked in G-Fix for 2 days and incubated in 70% ethanol for 1 day. The brains were embedded in paraffin on a CT-Pro 20 using G-Nox (GenoStaff) and cut into 8-μm sections. Sections were fixed with 10% neutral buffered formalin (NBF; 10% formalin in phosphate buffered formalin [PBS]) for 15 min at room temperature. Following this, the tissues were washed with PBS and treated with 4 μg/mL of proteinase K for 10 min at 37 °C. The tissues were fixed with 10% NBF at room temperature for 15 min and washed with PBS. They were then treated with 0.2 N hydrochloric acid at room temperature for 10 min and washed with PBS. Hybridization was conducted at 60 °C for 16 h in a G-Hybo-L solution with a probe at a concentration of 300 ng/mL. For the TNF-α probe, we used sequence position 117–689. The coloring reaction was conducted using a nitro blue tetrazolium/5-bromo-4-chloro-3-indolyl-phosphate solution. Immunostaining was performed with Iba1 (FUJIFILM Wako, Osaka, Japan) and GFAP antibodies (Cell Signaling Technology, Danvers, MA, USA) and detected by 3,3′-diaminobenzidine staining. Sections were counterstained with Kernechtrot stain and mounted with G-mount (GenoStaff).

### Cells and cell cultures

Mouse primary microglia were isolated from a litter of postnatal day (P) 2 pups, according to previous reports^73^. Briefly, mice were placed in an ice bath and dissected to isolate the cerebral cortices. The cerebral cortices were then minced in a 0.01% trypsin PBS solution and incubated at 37 °C for 15 min. Dulbecco’s modified Eagle’s medium (DMEM; supplemented with 10% fetal bovine serum [FBS], 0.3% L-glutamine, and antibiotics [100 U/mL penicillin and 100 μg/mL streptomycin]) was added and centrifuged (1,000 g for 5 min at 4 °C). The supernatants were discarded and the remaining pellet was suspended in DMEM (supplemented with 10% FBS, 0.3% L-glutamine, and antibiotics [100 U/mL penicillin and 100 μg/mL streptomycin]). The cell suspension was filtrated using a 40 µm cell strainer (Corning, NY, USA) and seeded on a 10 cm dish. The media were changed every 3 days and incubated for 15 days. Floating cells were collected as resting microglia and seeded on a poly-D-lysine-coated 12-well plate (1 × 10^5^ cells/well). After 24 h of incubation, the cells were used as mouse primary microglia. Mouse microglial cells (BV2) were cultured in DMEM (supplemented with 10% FBS, 0.3% L-glutamine, and antibiotics [100 U/mL penicillin and 100 μg/mL streptomycin]). The cells (2.5 × 10^5^ cells/well) were seeded on a poly-D-lysine-coated 12-well plate and exposed to methylmercury after 24 h of incubation. These cells were maintained at 37 °C in a humidified incubator in an atmosphere of CO_2_ (5%) and ambient air (95%).

### Transfection of siRNA

The BV2 cells (5 × 10^4^ cells/well for ASK1 or p65 knockdown; 1.25 × 10^5^ cells/well for TAK1 knockdown) were seeded on a poly-D-lysine-coated 12-well plate and cultured for 24 h. The indicated siRNA was then transfected with Lipofectamine RNAiMAX transfection reagent (Thermo Fisher Scientific, Waltham, MA, USA). For the transfection, we used 1.5 µL of Lipofectamine solution and 2 µL of siRNA (10 µM). The cells were cultured for a further 24 h for TAK1 knockdown or 48 h for ASK1 knockdown, then exposed to methylmercury for the indicated condition. All siRNAs were purchased from Sigma-Aldrich (St. Louis, MO, USA). Forward sequences of siRNA are listed below: TAK1 siRNA #1 (5′-CCAUUAUAACAGUUCAUGAdTdT-3′); TAK1 siRNA #2 (5′-CUAACAUUGUCAAGUUGUAdTdT-3′); ASK1 siRNA #1 (5′-CUGAGUAGCCUUCUGGGUAdTdT-3′); ASK1 siRNA #2 (5′-GCAGAUACUGGAAGGAUUAdTdT-3′); p65 siRNA #1 (5′-CUAUGAGACCUUCAAGACUdTdT-3′); and p65 siRNA #2 (5′-GAAGAAGAGUCCUUUGAAUdTdT-3′). Negative control siRNAs were also obtained from Sigma-Aldrich.

### Quantitative PCR (qPCR)

Total RNA was isolated using Isogen II (NIPPON GENE, Tokyo, Japan), according to the manufacturer instructions. Reverse transcription was performed using PrimeScript RT reagent kit (Takara-Bio, Shiga, Japan). Quantitative PCR was performed using KAPA SYBR (NIPPON Genetics, Tokyo, Japan) by Thermal Cycler Dice (Takara-Bio) with the following primers: *Tnf*, F:5′-CACACTCACAAACCACCAAGTG-3′, R:5′-TTTGAGATCCATGCCGTTGG-3′; *Actin*, F:5′-GGCTGTATTCCCCTCCATCG-3′, R:5′-CCAGTTGGTAACAATGCCATGT-3′; and glyceraldehyde 3-phosphate dehydrogenase (*Gapdh*), F:5′-AACTTTGGCATTGTGGAAGG-3′, R:5′-ACACATTGGGGGTAGGAACA-3′. Actin was used as an internal control for BV2 and GAPDH was used for primary microglia (because exposure of BV2 cells to methylmercury resulted in a decrease of GAPDH, we avoided the use of GAPDH as an internal control). The data are presented as values corrected for actin or GAPDH.

### Western blotting

The cells were harvested in a sodium dodecyl sulfate (SDS) buffer (1 mM trisaminomethane-hydrochloride [pH 7.4], 2% SDS, 150 mM sodium chloride [NaCl], and 1 mM ethylenediaminetetraacetic acid) supplemented with a protease inhibitor cocktail (Roche, Basel, Switzerland). The lysates were then incubated at 95 °C for 10 min. Protein concentration of each lysate was examined using the DC protein assay kit (Bio-Rad, Hercules, CA, USA). An aliquot of a sample (20 µg) was subjected to SDS–polyacrylamide gel electrophoresis. The gel was transferred to an Immobilon-P polyvinylidene fluoride membrane (Merck Millipore, Burlington, MA, USA) and stained with antibodies against TAK1, phospho-JNK, JNK, phospho-p38 (P-p38), p38, phospho-ERK, ERK, phospho-p65 (Cell Signaling Technology), ASK1, GAPDH, and Iba1 (FUJIFILM Wako). NeuN antibodies were obtained from Abcam (Cambridge, UK). Uncropped data of Figs. 3, 4, 5 and 6 are shown in supplemental data (Supplemental Fig. 8–11).

### Measurement of intracellular ROS

The cells were incubated with 10 µM of H_2_DCF-DA (Thermo Fisher Scientific) or 1 µM of Mito-SOX Red Mitochondrial Superoxide Indicator (Mito-SOX) (Thermo Fisher Scientific) for 30 min. Following this, the cells were exposed to methylmercury for the indicated condition. The cells were rinsed with PBS and the medium was changed to Hanks' balanced salt solution (HBSS). Fluorescence was detected by the plate reader, SpectraMax Gemini XPS (Molecular Devices, San Jose, CA, USA), set at Ex 490 nm/Em 530 nm (H_2_DCF-DA) or Ex 510 nm/Em 580 nm (Mito-SOX).

### Organotypic cerebral slice culture preparation

Mouse brain slice cultures were prepared according to our previous report^73^. The cerebral cortices of the mouse pups (P7) were dissected and immediately placed in ice-cold HBSS, containing 6 mg/mL of glucose and 15 mM of 4-(2-hydroxyethyl)-1-piperazineethanesulfonic acid (HEPES) for 5 min. The cerebral cortices were cut into 350 µm-thick sagittal slices using a McIlwain Tissue Chopper (Mickle Laboratory Engineering, Cambridge, UK) and incubated on ice for 30 min in HBSS, containing 6 mg/mL of glucose and 15 mM of HEPES. Two intact slices were placed on the culture plate insert of the 6-well plate and maintained in 5% CO_2_ at 37 °C. The incubation media were replaced after 24 h and used for after 3 days.

### Statistical analysis

Statistical significance was analyzed using a one-way ANOVA and Tukey’s post hoc test. Analyses were performed with GraphPad Prism 8 (GraphPad Software, CA, USA).

### Ethical approval

All animal experiments were approved by ethics committee of Tohoku University.

### Consent to participate

All authors checked the study and agreed to participate in the manuscript.

### Consent for publication

All authors agreed to the publication.

## Supplementary Information

Supplementary Information

## Data Availability

All data needed to evaluate the conclusions in the paper are present in the paper or the Supplementary Materials.
