# Supplementary material for: Methylmercury induces neuronal cell death by inducing TNF-α expression through the ASK1/p38 signaling pathway in microglia
Source: Sci Rep. 2021 May 10;11:9832. doi: 10.1038/s41598-021-89210-7 (PMC8110582; doi:10.1038/s41598-021-89210-7)
Supplement: Supplementary file 1 — Supplementary Information [file 41598_2021_89210_MOESM1_ESM.pdf]

Supplemental information

Methylmercury induces neuronal cell death by inducing TNF-α expression through the ASK1/p38 signaling pathway in microglia

Takashi Toyama[1,2], Takayuki Hoshi[1,2,3], Takuya Noguchi[4], Yoshiro Saito[2], Atsushi Matsuzawa[4], Akira Naganuma[1] and Gi-Wook Hwang[1,3,*]

[1] *Laboratory of Molecular and Biochemical Toxicology, Graduate School of Pharmaceutical Sciences, Tohoku University, 6-3 Aoba, Aramaki, Aoba-ku, Sendai, Miyagi 980-8578, Japan*

[2] *Laboratory of Molecular Biology and Metabolism, Graduate School of Pharmaceutical Sciences, Tohoku University, 6-3 Aoba, Aramaki, Aoba-ku, Sendai, Miyagi 980-8578, Japan*

[3] *Laboratory of Environmental and Health Sciences, Faculty of Pharmaceutical Sciences, Tohoku Medical and Pharmaceutical University, 4-4-1 Komatsushima, Aoba-ku, Sendai, Miyagi 981-8558, Japan*

[4] *Laboratory of Health Chemistry, Graduate School of Pharmaceutical Sciences, Tohoku University, 6-3 Aoba, Aramaki, Aoba-ku, Sendai, Miyagi 980-8578, Japan*

Running title: A mechanism of TNF-α induction by methylmercury

[*]Correspondence and requests for materials should be addressed to:

Gi-Wook Hwang, Ph.D.

Professor

Laboratory of Environmental and Health Sciences, Faculty of Pharmaceutical Sciences, Tohoku Medical and Pharmaceutical University, 4-4-1 Komatsushima, Aoba-ku, Sendai, Miyagi 981-8558, Japan

Phone: +81-22-727-0133

E-mail: hwang@tohoku-mpu.ac.jp

Materials and methods

Measurement of mercury concentration

Mercury concentrations in the brain (approximately 3–5 mg for each tissue) were measured with a heated vaporized atomic absorption spectrometer (Zeeman Mercury Spectrometer RA-915^{+}, Lumex Ltd., St. Petersburg, Russia). A standard curve was prepared with an inorganic mercury standard solution.

Transfection of siRNA

BV2 cells (5×10^4 cells/well) were seeded on a poly-*D*-lysine-coated 12-well plate and cultured for 24 hours. Then, the indicated siRNA was transfected with Lipofectamine RNAiMAX transfection reagent (Thermo Fisher Scientific, Waltham, MA, USA). Sequences of siRNA were as follows: c-Jun siRNA #1 (5′-GUGCCUACGGCUACAGUAA-3′); c-Jun siRNA #2 (5′-CAGUAACCCUAAGAUCCUA-3′); c-Fos siRNA#1 (5′-GAAUCCGAAGGGAACGGAA-3′); and c-Fos siRNA #2 (5′-GAUACACUCCAAGCGGAGA-3′).

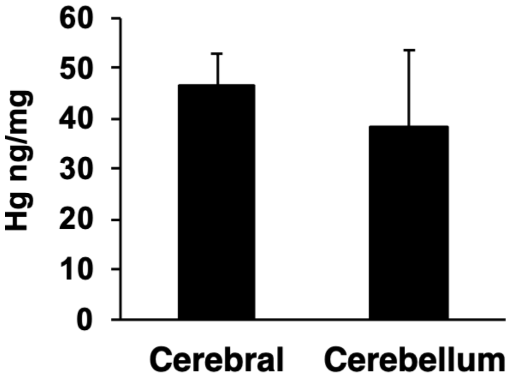

Supplemental Fig. 1. Mercury concentrations in the brains of mice administrated with methylmercury

Mice were injected with MeHgCl (25 mg/kg) by S.C. and kept for 7 days *ad libitum*. The mercury concentration in the mouse brain was subjected to an atomic absorption spectrometer (n = 5). The data are represented as mean ± standard deviation of total mercury content (ng of Hg), corrected for the wet weight of the brain (mg of brain).

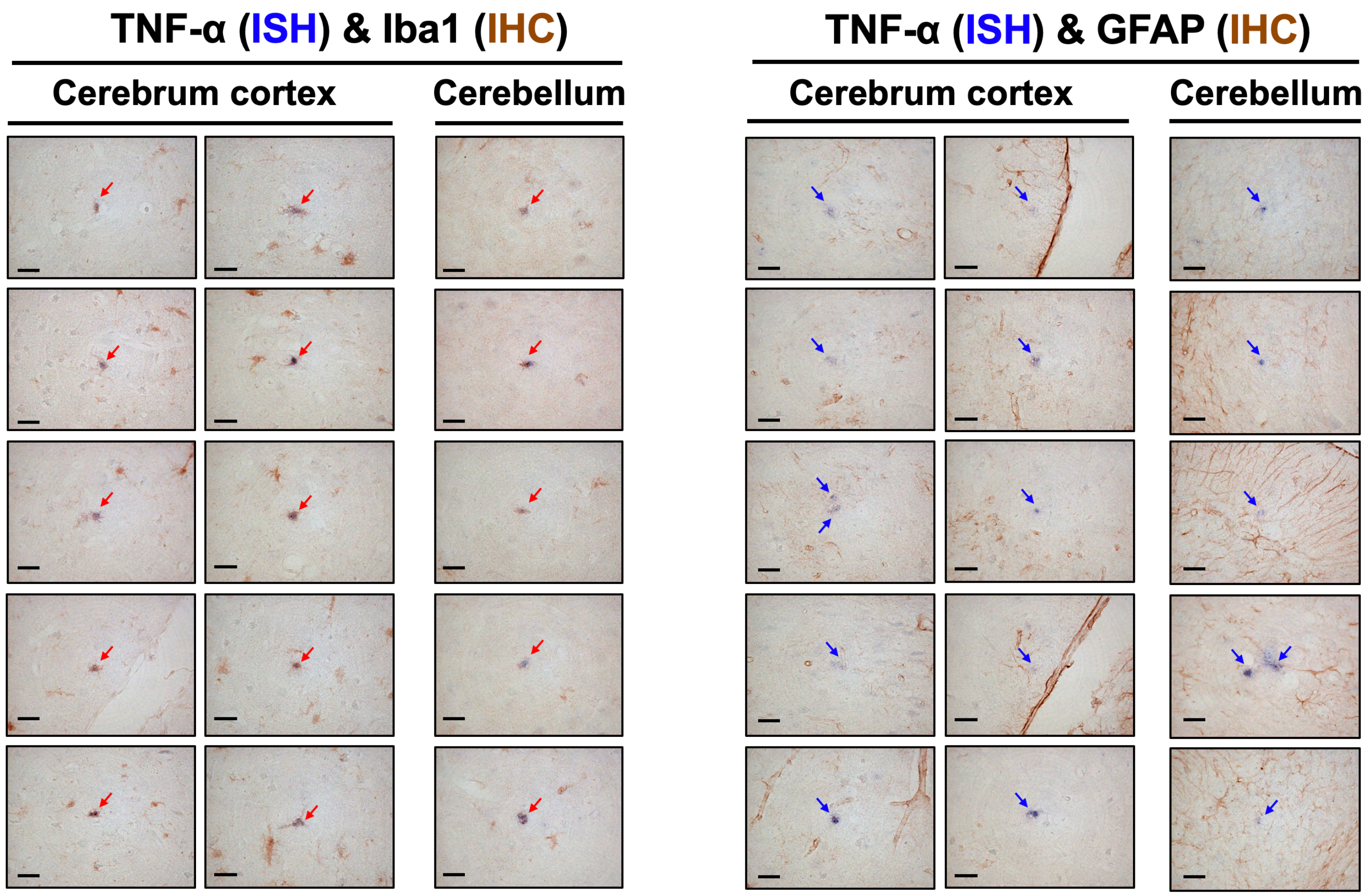

Supplemental Fig. 2. Methylmercury-induced TNF-α expressing cells in the brains of mice

Mice were injected with MeHgCl (25 mg/kg) by S.C. After 7days the brains were subjected to in situ hybridization for TNF-α mRNA (blue spots). The section was immunostained for GFAP or Iba1 antibodies. Red arrows indicate double positive cells of TNF-α and immunostainings. Blue arrows indicate ISH positive cells. Scale bars indicate 25 μm.

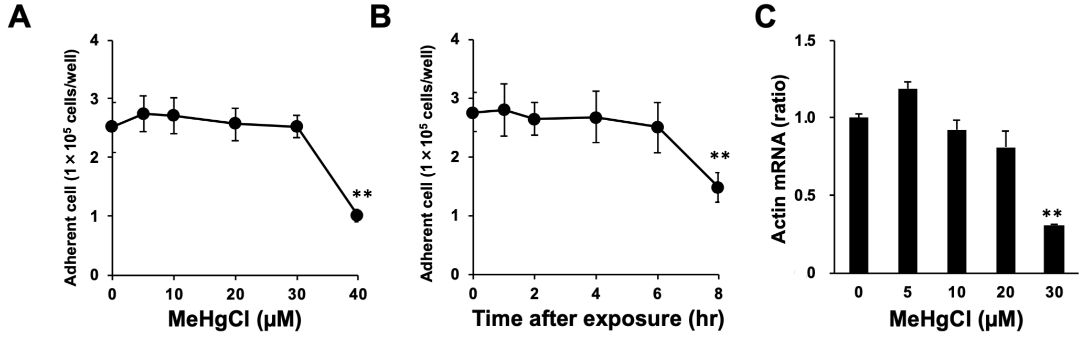

Supplemental Fig. 3. Effects of methylmercury on mRNA levels of actin in BV2 cells

(a) BV2 cells were exposed to indicated concentrations of MeHgCl for 6 hours or 20 μM MeHgCl for the indicated time course (b), and adherent cells were counted (n = 3). (c) mRNA levels of actin were measured by qPCR (n = 3). The data are presented as mean ± standard deviation (n = 3). The Y-axis indicates the ratio with the control as 1. $^{**}P < 0.01$ vs control.

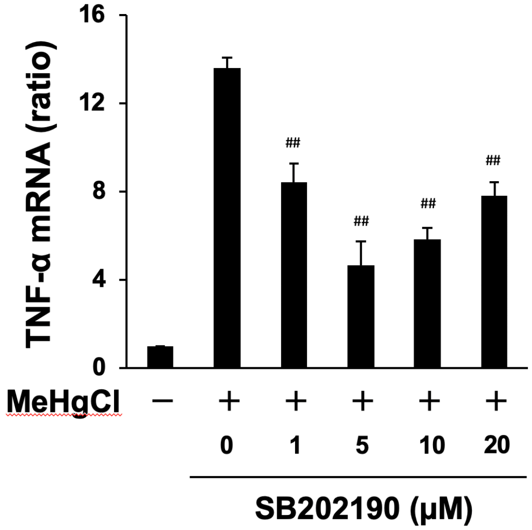

Supplemental Fig. 4. Effects of SB202190 on methylmercury-induced TNF-α expression

The cells were pretreated with indicated concentrations of SB202190 (p38 inhibitor) for 30 minutes, then 20 μM of MeHgCl were added to the medium and incubated for a further 6 hours. mRNA levels of TNF-α were measured by qPCR (n = 3). The data are shown as actin-corrected means ± standard deviations. The Y-axis indicates the ratio with the control as 1. $^{\#} < 0.05$ vs MeHgCl(+), $^{\#\#} < 0.01$ vs MeHgCl(+).

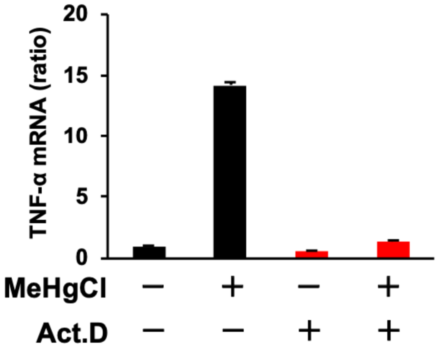

Supplemental Fig. 5. Effects of actinomycin D on methylmercury-induced TNF-α expression

The cells were treated with actinomycin D and 20 μM of MeHgCl for 6 hours. mRNA levels of TNF-α were measured by qPCR (n = 3). The data are shown as actin-corrected means ± standard deviations. The Y-axis indicates the ratio with the control as 1.

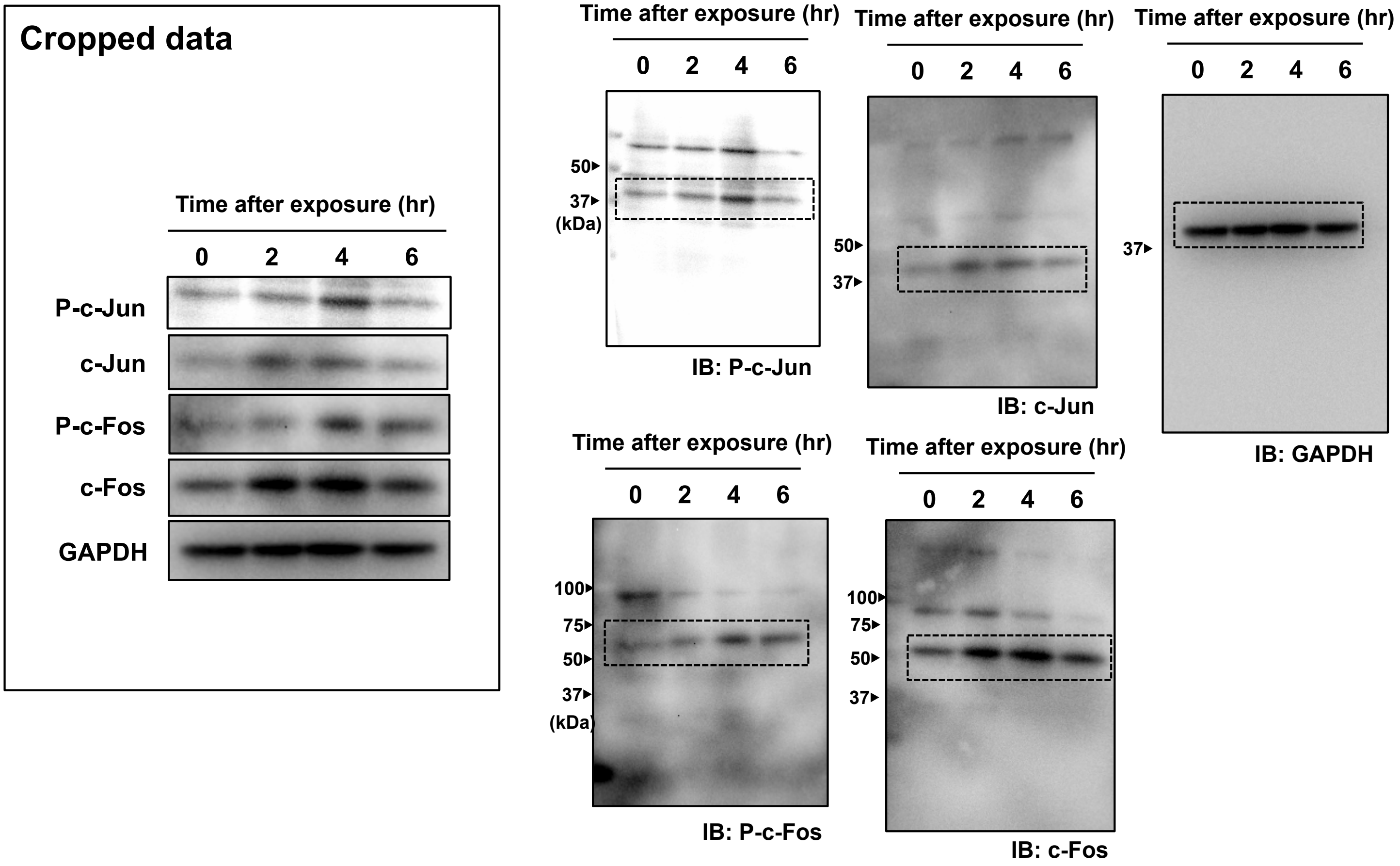

Supplemental Fig. 6. Effects of methylmercury on the phosphorylation of c-Jun and c-Fos

The cells were treated with 20 µM of MeHgCl for the indicated time and Western blotting was performed.

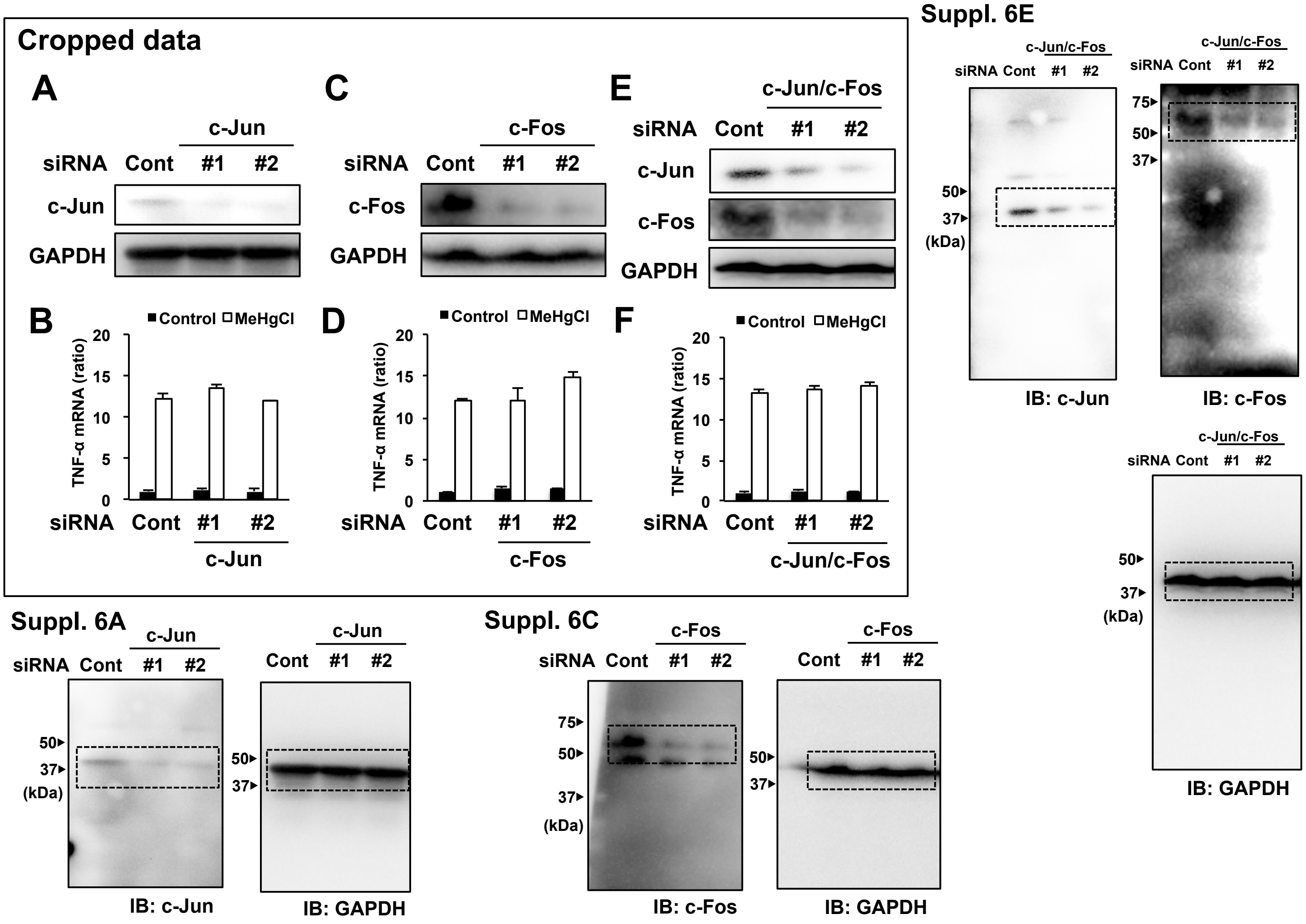

Supplemental Fig. 7. Effects of c-Jun and c-Fos on methylmercury-induced TNF-α expression

BV2 cells were transfected with 2 different sequences of c-Jun or c-Fos siRNA (#1 and #2) or c-Jun and c-Fos siRNA (each siRNA #1 + #1 or #2 + #2, respectively) and incubated for 24 hours. Protein levels of (a) c-Jun, (c) c-Fos, or (e) both were determined by Western blotting. (b, d, f) The cells were exposed to MeHgCl (20 μM) for 6 hours and mRNA levels of TNF-α were measured by qPCR (n = 3). The data are shown as actin-corrected means ± standard deviations. The Y-axis indicates the ratio with the control as 1.

Fig. 3A

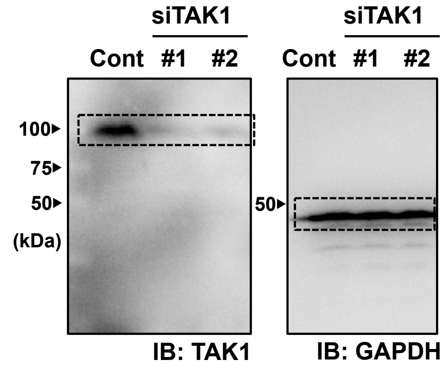

Fig. 3C

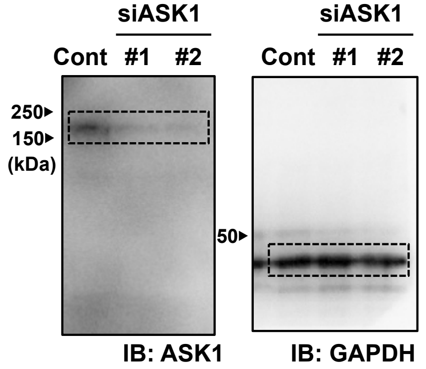

Fig. 3E

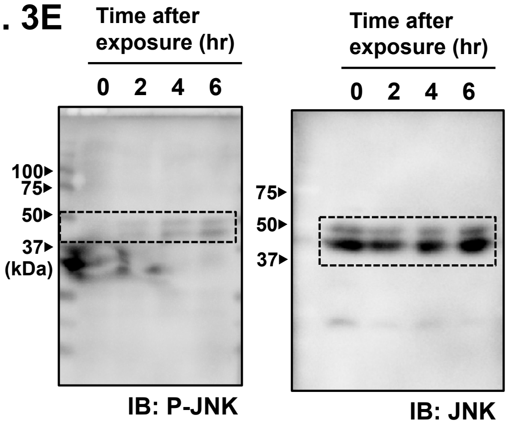

Fig. 3E

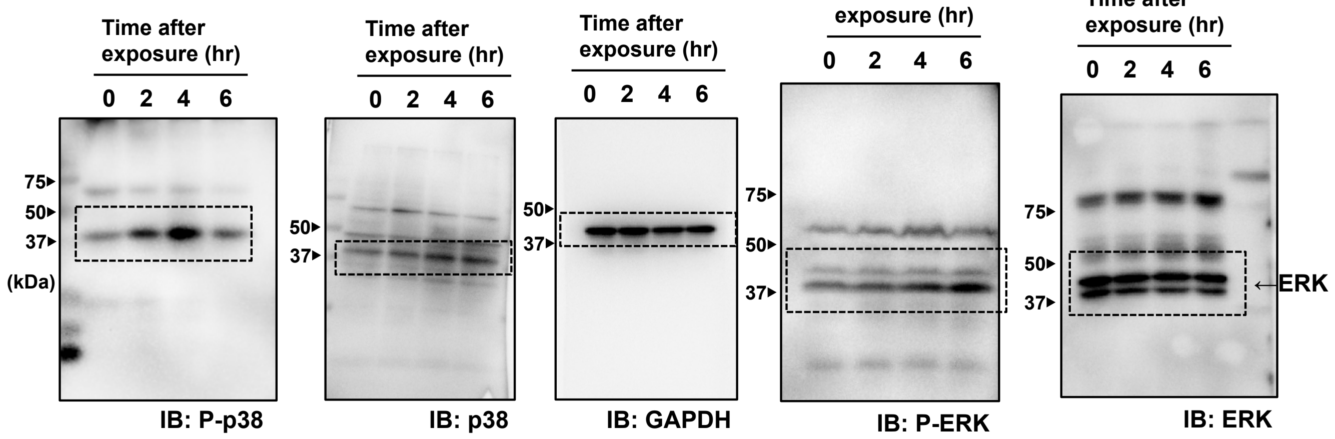

Fig. 3G

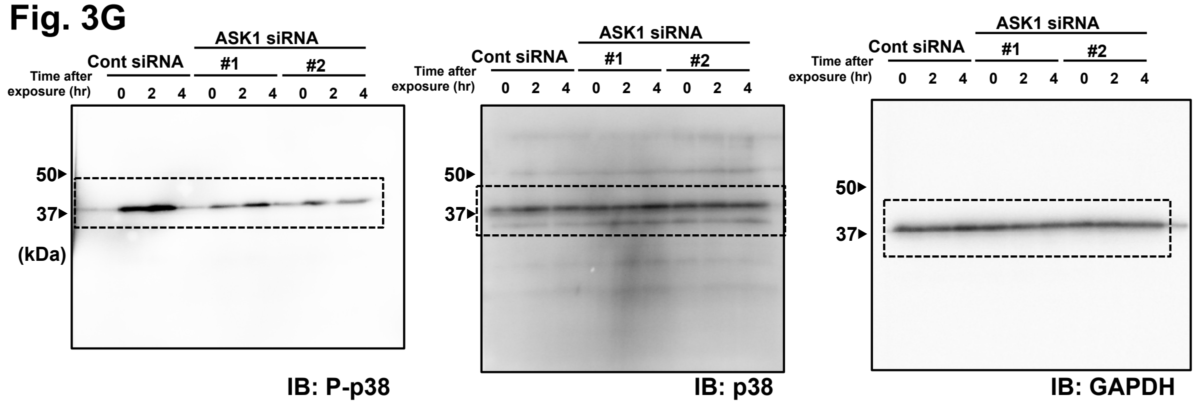

Supplemental Fig. 8. Uncropped data of figure 3.

Supplemental figure 8

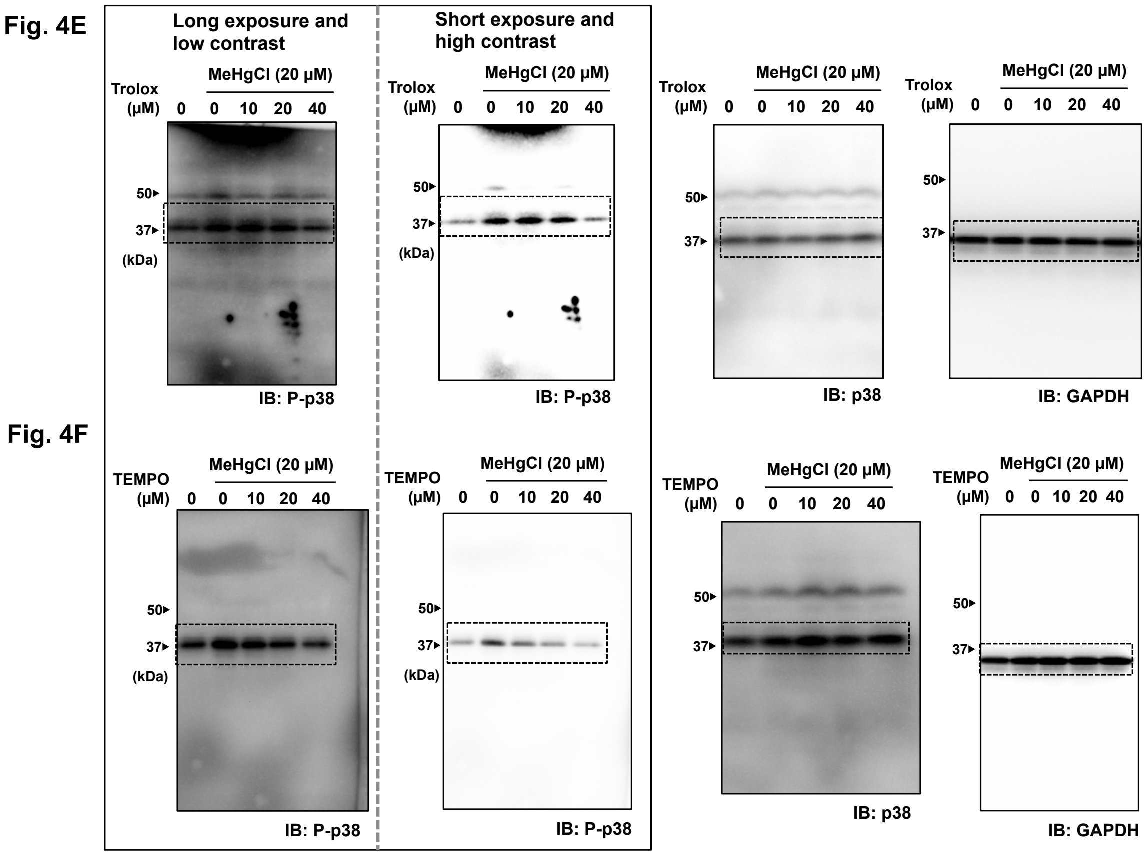

Supplemental Fig. 9. Uncropped data of figure 4.

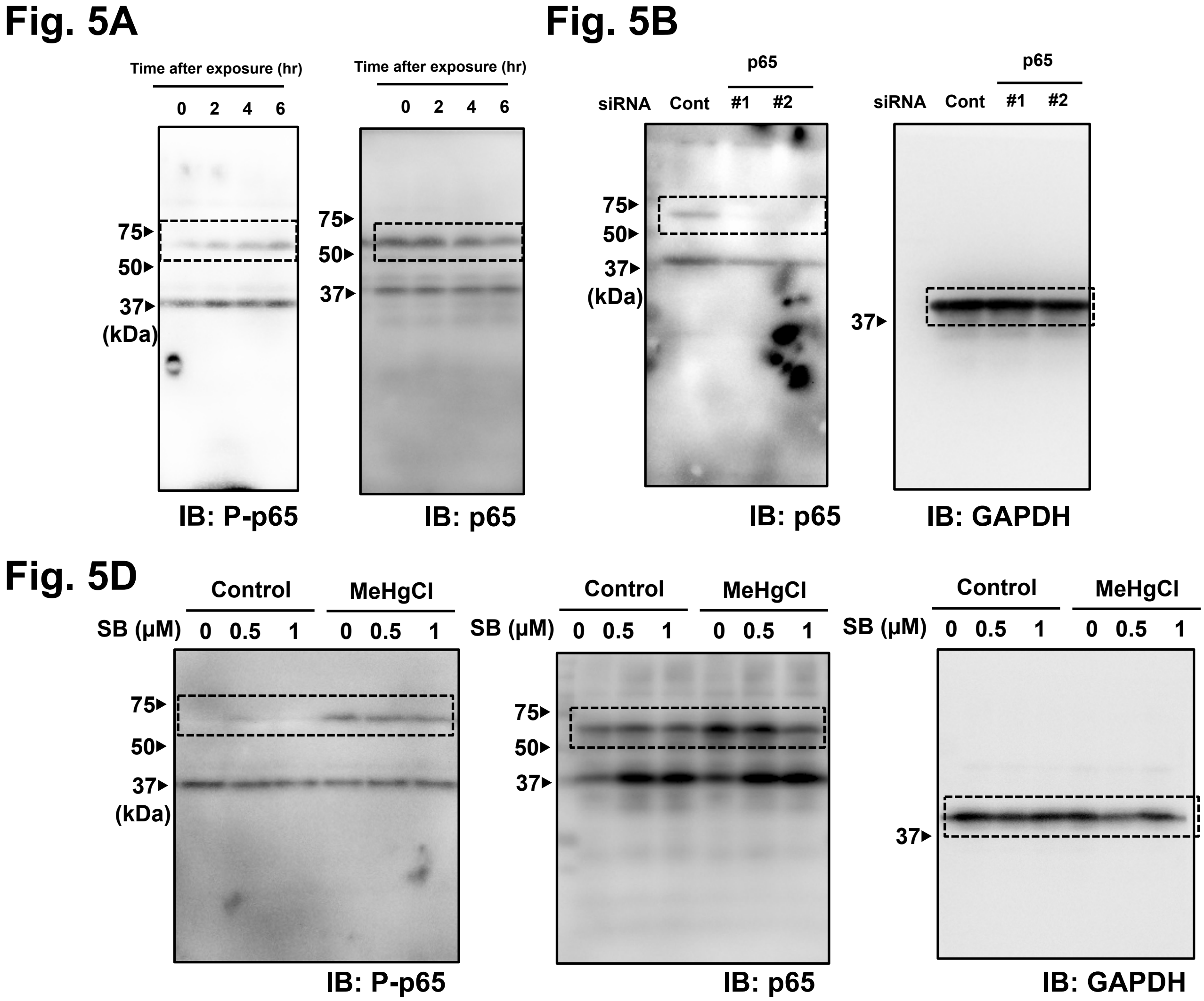

Supplemental Fig. 10. Uncropped data of figure 5.

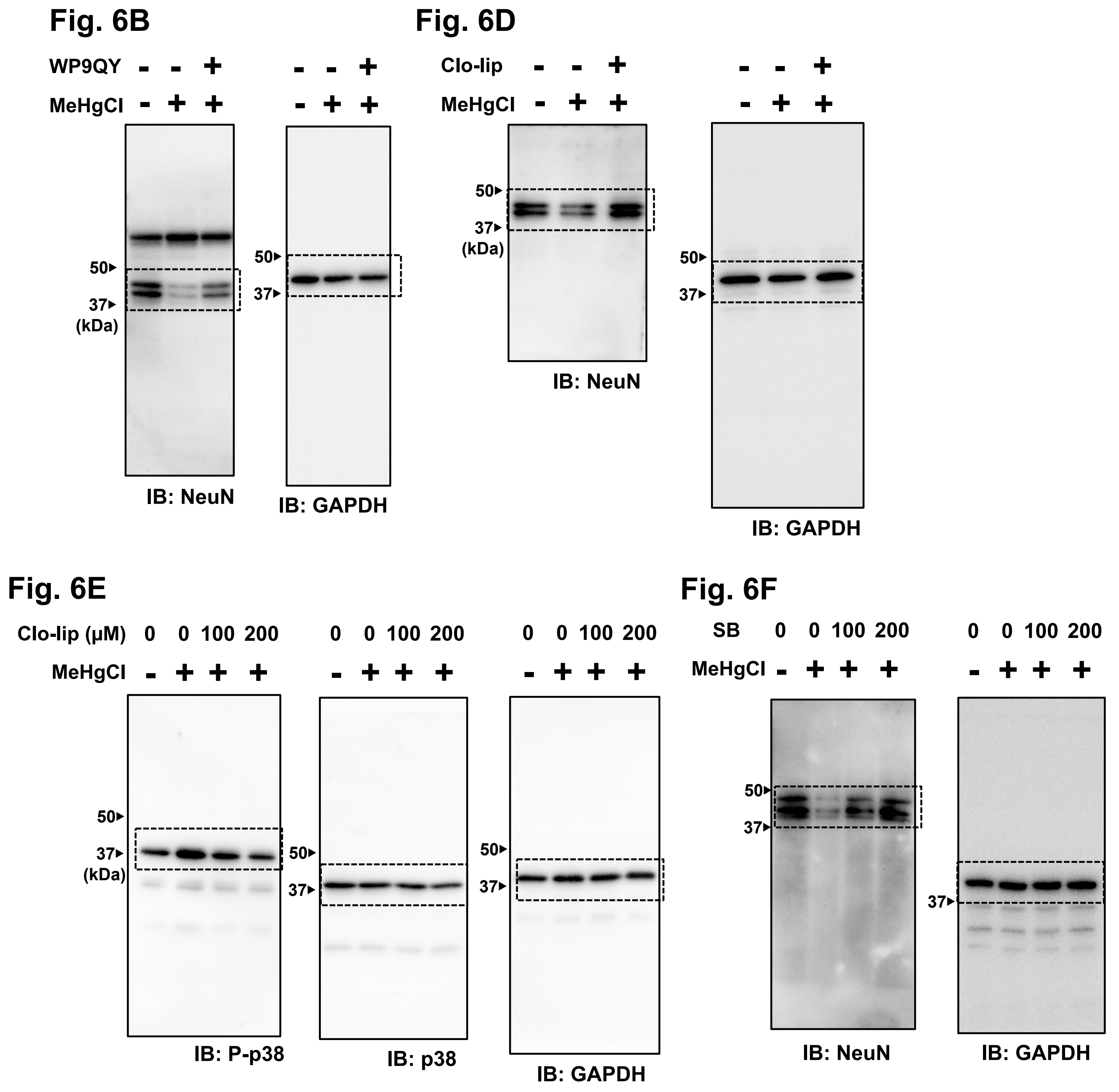

Supplemental Fig. 11. Uncropped data of figure 6.
